# Two new species of *Stenochironomus* Kieffer (Diptera, Chironomidae) from Zhejiang, China

**DOI:** 10.3897/zookeys.479.8364

**Published:** 2015-01-29

**Authors:** Xin Qi, Xiaolong Lin, Yuedan Liu, Xinhua Wang

**Affiliations:** 1 College of Life Science, Taizhou University, Taizhou, Zhejiang 318000, China; 2 College of Life Science, Nankai University, Tianjin 300071, China; 3 The Key Laboratory of Water and Air Pollution Control of Guangdong Province, South China Institute of Environmental Sciences, Ministry of Environment Protection of PRC, Guangzhou 510065, China; 4 State Environmental Protection Key Laboratory of Water Environmental Stimulation and Pollution Control, South China Institute of Environmental Sciences, Ministry of Environment Protection of PRC, Guangzhou 510065, China

**Keywords:** *Stenochironomus*, new species, key, China

## Abstract

Two new species of *Stenochironomus* Kieffer (Diptera: Chironomidae: Chironominae), *Stenochironomus brevissimus* **sp. n.** and *Stenochironomus linanensis* **sp. n.**, are described from China and the male imagines are illustrated. *Stenochironomus brevissimus* **sp. n.** can be separated from the so far known species by having very short and small, spatulate superior volsella with two long setae, whereas *Stenochironomus linanensis* **sp. n.** is easily separated from the other species of *Stenochironomus* by the following characters: wings transparent, body yellow, superior volsella finger-like, with nine long setae, elongated inferior volsella with four long setae and one well developed terminal spine; tergite IX with 10−15 long setae medially. A key to the males of *Stenochironomus* occurring in China is given.

## Introduction

*Stenochironomus* Kieffer, 1919 is a species-rich genus with worldwide distribution, occurring in all biogeographical regions except in Antarctica (Cranston et al. 1989). The genus was erected by Townes in 1945 based on *Chironomus pulchripennis* (Coquillett, 1902) (Spies and Sæther 2004). The larvae are easily found mining decayed leaves or wood in freshwater habitats ranging from small ponds and swamps to fast-flowing streams and rivers (Cranston et al. 1989). Based on the different hosts of larvae and pupae, Borkent (1984) erected two subgenera: *Stenochironomus* *s. str.* Kieffer (larvae and pupae mine dead submerged wood) and *Petalopholeus* Borkent (larvae and pupae mine dead submerged leaves). This subdivision has not been adopted by subsequent authors, because immature stages are known only for a few species, which makes difficult to ascribe them to any subgenus (Pinho et al. 2005, Andersen et al. 2008, Qi et al. 2008, Dantas et al. 2010, Zorina 2010). To date, there are 97 species recorded around the word: 24 species from the Palaearctic Region, 17 from the Nearctic Region, 30 from the Neotropical Region, 16 from the Oriental Region, 16 from the Afrotropical Region and 4 from the Australasian Region (Qi et al. 2011, Reis et al. 2013).

Zhejiang Province is located in the Chinese central subtropical region, which has a humid monsoon climate. In Zhejiang, three species of *Stenochironomus* [*Stenochironomus koreanus* Borkent, 1984, *Stenochironomus nubilipennis* Yamamoto, 1981 and *Stenochironomus satorui* (Tokunaga & Kuroda, 1936)] have been recorded (Wang 2000, Qi et al. 2011). In this paper, two new species of *Stenochironomus* from Zhejiang, Oriental China, are described and illustrated. A key to the males of *Stenochironomus* from China is presented.

## Materials and methods

The morphological nomenclature follows Sæther (1980). Measurement methods follow Qi et al. (2012). The material examined was slide-mounted, following the procedure outlined by Sæther (1969). Specimens have been deposited in the College of Life Science, Taizhou University, China.

Abbreviations of parts measured are as follows:

AR Antennal ration, length of 13^th^ / length of flagellomeres 1–12

Palpomere ratio (5th/3rd) Length of the 5^th^ Palpomere / length of the 3^rd^ Palpomere

VR Venarum ration, length of Cubitus (Cu) / length of Media (M)

BV Length of (femur + tibia + ta_1_) / length of (ta_2_ + ta_3_ + ta_4_ + ta_5_)

LR Leg ration, length of ta_1_ / length of tibia

SV Length of (femur + tibia) / length of ta_1_

HR Hypopygium ration, length of gonocoxite / length of gonostylus

HV Hypopygium value, total length / length of gonostylus times ten

p1 Fore leg

p2 Mid leg

p3 Hind leg

fe femur

ti tibia

ta1…tan tarsus_1_…tarsus_n_

R Radius

R1 Radius 1 vein

R4+5 Radius 4+5 vein

## Taxonomy

### Stenochironomus brevissimus sp. n.

Taxon classificationAnimaliaDipteraChironomidae

http://zoobank.org/ADC155AF-0069-4052-8907-191E35A59854

Figs 1–10

#### Diagnosis.

The adult male of *Stenochironomus brevissimus* sp. n. can be distinguished from all other species of *Stenochironomus* by the following combination of characters: superior volsella very short and small, spatulate, with 2 long setae, elongated inferior volsella with 6 long setae, posterior margin of tergite IX with 20−22 setae and 8 spines.

#### Description.

Male imago (n = 3). Total length 4.3−4.6 mm. Wing length 2.2−2.5 mm. Total length / wing length 1.8−1.9. Wing length / length of profemur 1.7−1.8.

Coloration. Head yellow, antenna brown. Thorax light yellow, postnotum and scutum with brown spots. Wings transparent, without any pigmentation. Abdomen and hypopygium yellow, anal point brown. Fore legs yellow with femur apically brown. Mid legs with apex and posterior basal region of femur with dark brown stripes, tibia brown, tarsomeres 1–5 yellow. Hind legs brown with femur yellow with dark brown stripes at apex.

**Figures 1–10.**
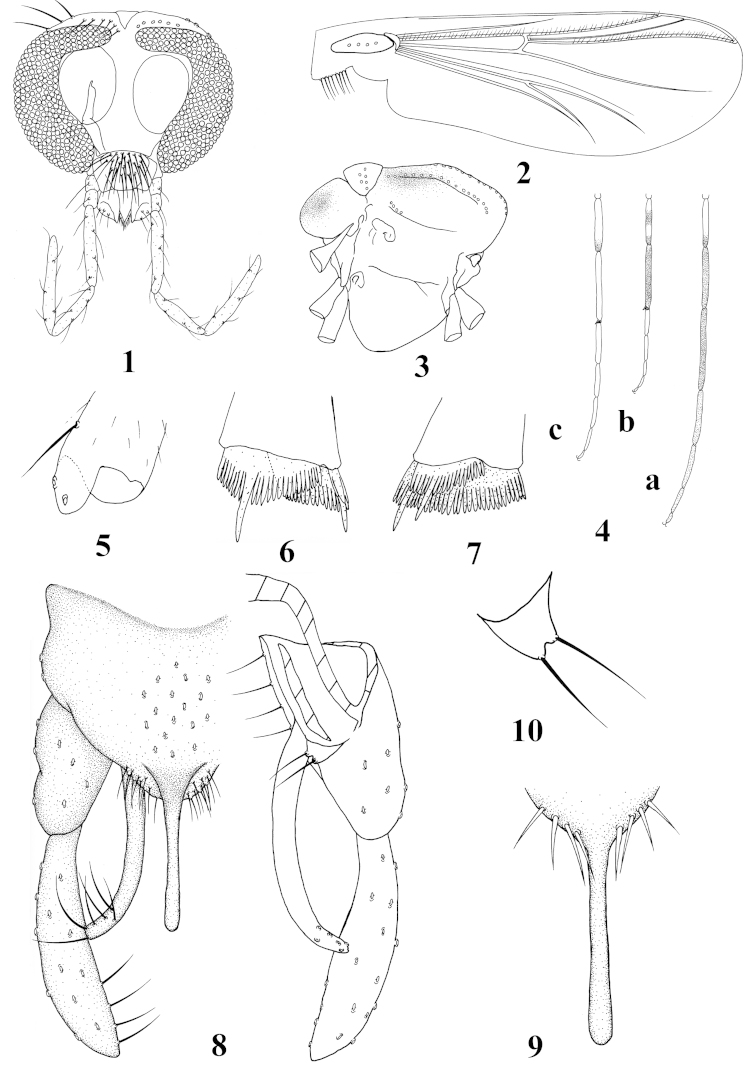
*Stenochironomus brevissimus* sp. n., male. **1** head **2** wing **3** thorax, lateral view **4** legs coloration (a. fore leg; b. mid leg; c. hind leg) **5** fore tibial apex, ventral view **6** mid tibial apex, lateral view **7** hind tibial apex, lateral view **8** hypopygium **9** spines on posterior margin of tergite IX, ventral view **10** superior volsella.

Head (Fig. 1). AR 1.80−1.92. Temporal with 10−12 setae. Clypeus with 20−22 setae. Tentorium 173−176 mm long, 43−46 mm wide. Stipes 140−145 µm long, 10−13 µm wide. Palpomere lengths (in mm): 60−63, 60−65, 210−230, 140−150, 260−300. Palpomere ratio (5^th^/3^rd^) 1.2−1.3.

Wings (Fig. 2). VR 1.08−1.15. Brachiolum with 3−4 setae; R with 25−32 setae, R_1_ with 27−30 setae, R_4+5_ with 41−42 setae. Squama with 8−10 setae.

Thorax (Fig. 3). Dorsocentrals 12−14, acrostichals 14−16, prealars 4−5. Scutellum with 6−7 setae.

Legs (Fig. 4). Fore leg: width at apex of tibia 60−65 mm, tibia with scale 53−56 µm long, with 2−4 strong setae (Fig. 5). Mid leg: width at apex of tibia 80−83 mm, tibia with two apical spurs 40−50, 43–53 μm long. Hind leg: width at apex of tibia 70−80 mm, tibia with two apical spurs 40−50, 40–55 μm long. Mid and hind tibiae with fused combs (Figs 6–7), each comb 36−50 µm long. Lengths (in mm) and proportions of legs in Table 1.

**Table 1.** Lengths (in µm) and proportions of leg segments in *Stenochironomus brevissimus* sp. n. (n = 3).

	p_1_	p_2_	p_3_
Fe	1300−1400	1125−1200	1325−1525
Ti	1300−1500	1000−1125	1275−1400
ta_1_	1500−1600	725−800	950−1100
ta_2_	775−875	375−450	525−625
ta_3_	675−750	340−410	440−500
ta_4_	525−600	200−290	260−360
ta_5_	250−300	90−110	110−130
LR	1.07−1.15	0.71−0.73	0.74−0.79
BV	1.78−1.84	2.48−2.83	2.49−2.66
SV	1.73−1.81	2.91−2.95	2.66−2.74

Hypopygium (Fig. 8). Anal point 113−120 mm long, 15−20 µm wide at base, 13−15 µm wide at apex, parallel-sided, slender, apically rounded. Tergite IX with 16−17 long setae medially, posterior margin of tergite IX with 20−22 setae and 8 spines (Fig. 9). Phallapodeme 120−123 mm long; transverse sternapodeme 30−50 mm long. Gonocoxite 200−230 mm long. Superior volsella short, small and spatulate, 16−18 mm long, 20−22 mm wide, with 2 long setae (Fig. 10). Inferior volsella elongated, 190−200 mm long, with 6 long setae. Gonostylus 210−230 mm long, with 4 long setae along inner margin in distal 1/3. HR 0.95−1.00, HV 1.89−2.04.

#### Female, pupa and larva.

Unknown.

#### Type material.

Holotype: Male, CHINA, Zhejiang, Quzhou City, Hunan County, 19.iv.2012, leg. XL Lin, sweep net. 2 Paratypes: 2 males, same data as holotype.

#### Etymology.

The specific epithet is a Latin adjective “*brevissimus*”, meaning the shortest, and refers to the superior volsella, which is the shortest in the genus.

#### Remarks.

*Stenochironomus brevissimus* sp. n. is similar to *Stenochironomus hainanus* Qi, Shi & Wang, 2008 and *Stenochironomus okialbus* Sasa, 1990 in having short and small superior volsella, but can separated from these species by the differences given in Table 2.

**Table 2.** Main differences between *Stenochironomus brevissimus* sp. n., *Stenochironomus hainanus* and *Stenochironomus okialbus*.

	*Stenochironomus brevissimus*	*Stenochironomus hainanus*	*Stenochironomus okialbus*
Wing	transparent, without any pigmentation	transparent, without any pigmentation	with dark bands across the middle and posterior area
Coloration	thorax light yellow, postnotum and scutum with brown spots; fore legs yellow with femur apically brown; mid legs with apex and posterior basal region of femur with dark brown stripes, tibia brown, tarsomeres 1–5 yellow; hind legs brown with femur yellow with dark brown stripes at apex	whole body yellow, without dark pigmentation	thorax yellow; fore legs yellow with femur apically brown, apex and basal region of tibia with dark brown stripes; mid legs yellow with femur apically brown; hind legs yellow with femur apically brown
Superior volsella	with 2 setae	with 3 setae	with 4 setae
Inferior volsella	with 6 setae	with 3 setae	with 4 setae and a strong terminal spine
Posterior margin of tergite IX	with 20−22 setae and 8 spines	with 16 setae	with 8 setae and 8 spines

#### Distribution.

The species is currently known only from Zhejiang Province of Oriental China.

### Stenochironomus linanensis sp. n.

Taxon classificationAnimaliaDipteraChironomidae

http://zoobank.org/F01888A4-0CA1-4041-9290-12B68CFA5BA0

Figs 11–18

#### Diagnosis.

The adult male of *Stenochironomus linanensis* sp. n. can be distinguished from all other species of *Stenochironomus* by the following combination of characters: wings transparent, body yellow, superior volsella finger-like, with 9 long setae, elongated inferior volsella with 4 long setae and one well-developed terminal spine, tergite IX with 10−15 long setae medially.

#### Description.

Male imago (n = 5). Total length 2.9−3.8 mm. Wing length 1.4−1.5 mm. Total length / wing length 1.98−2.41. Wing length / length of profemur 1.30−1.57.

Coloration. Head yellow. Thorax greenish yellow. Wings transparent, without any pigmentation. Abdomen yellow, hypopygium brown. Legs pale yellow.

**Figures 11–18.**
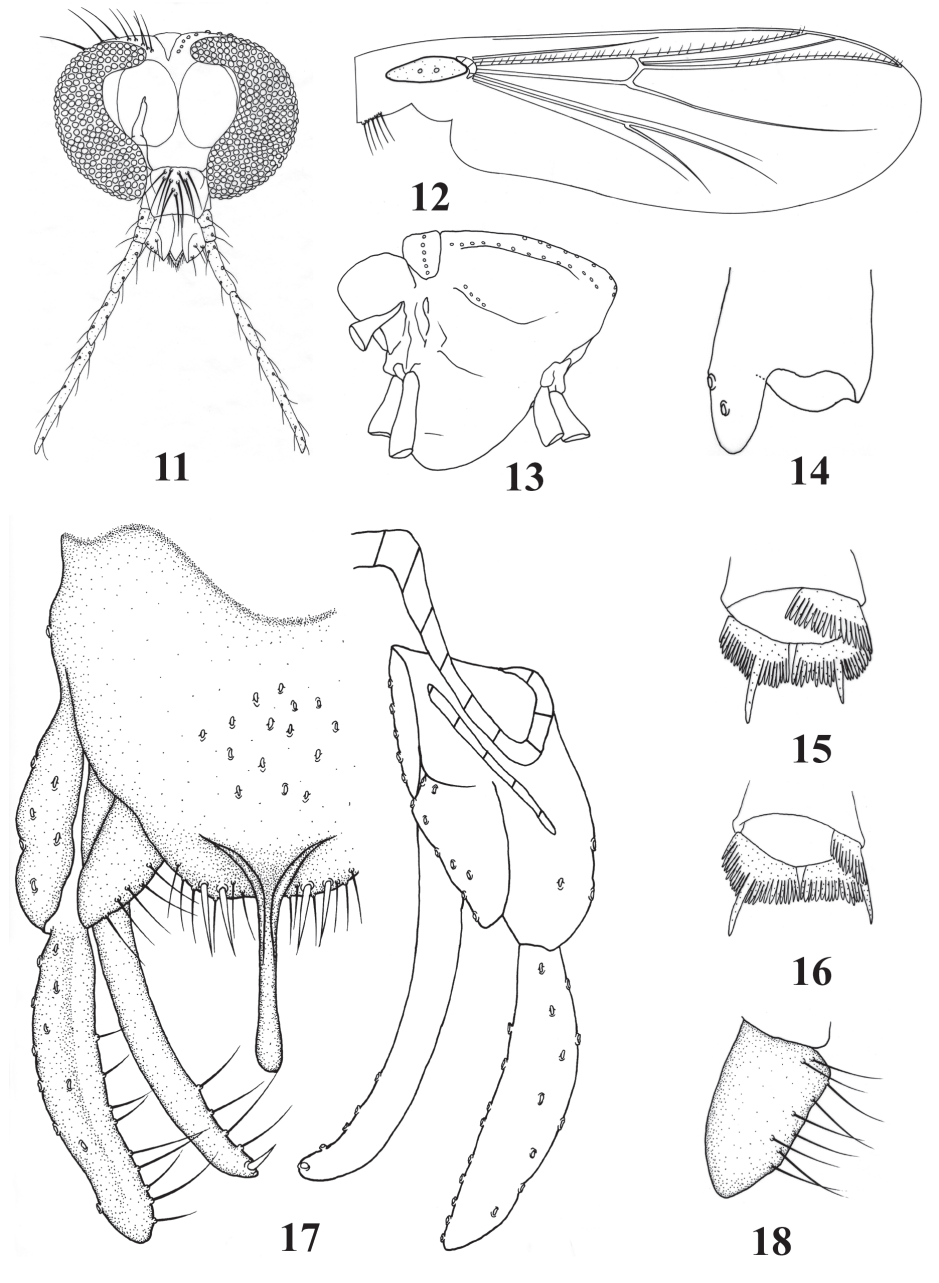
*Stenochironomus linanensis* sp. n., male. **11** head **12** wing **13** thorax, lateral view **14** fore tibial apex, ventral view **15** mid tibial apex, lateral view **16** hind tibial apex, lateral view **17** hypopygium **18** superior volsella.

Head (Fig. 11). AR 1.20−1.32. Temporal with 8−14 setae. Clypeus with 9−10 setae. Tentorium 153−156 mm long, 37−42 mm wide. Stipes 72−95 µm long, 5−6 µm wide. Palpomere lengths (in mm): 45−47, 28−33, 53−60, 75−90, 110−120. Palpomere ratio (5^th^/3^rd^) 2.00−2.07.

Wings (Fig. 12). VR 1.07−1.20. Brachiolum with 2 setae; R with 16−23 setae, R_1_ with 17−18 setae, R_4+5_ with 22−28 setae. Squama with 5−7 setae.

Thorax (Fig. 13). Dorsocentrals 9−13, acrostichals 9−14, prealars 4−5. Scutellum with 5−6 setae.

Legs. Fore leg: width at apex of tibia 33−47 mm, tibia with scale 33−46 µm long, with 2−3 strong setae (Fig. 14). Mid leg: width at apex of tibia 50−65 mm, tibia with two apical spurs 25−28, 30–40 μm long. Hind leg: width at apex of tibia 50−60 mm, tibia with two apical spurs 32−39, 35–40 μm long. Mid and hind tibiae with fused combs (Fig. 15–16), each comb 18−22 mm long. Lengths (in mm) and proportions of legs in Table 3.

**Table 3.** Lengths (in µm) and proportions of leg segments in *Stenochironomus linanensis* sp. n.

	p_1_	p_2_	p_3_
fe	925−1075	625−700	650−725
ti	700−875	650−725	750−900
ta_1_	925−1100	475−550	780−950
ta_2_	500−725	200−260	400−600
ta_3_	400−500	210−300	300−350
ta_4_	320−400	130−200	240−300
ta_5_	140−200	70−80	100−120
LR	1.25−1.32	0.73−0.75	1.04−1.06
BV	1.67−1.88	2.35−2.87	1.88−2.10
SV	1.76−1.83	2.60−2.68	1.71−1.79

Hypopygium (Fig. 17). Anal point 65−73 mm long, 14−20 µm wide at base, 6−8 µm wide at apex, apex of anal point slightly swollen and rounded. Tergite IX with 10−15 long setae medially, posterior margin of tergite IX with 12−14 setae and 4 spines. Phallapodeme 70−80 mm long; transverse sternapodeme 35−38 mm long. Gonocoxite 160−180 mm long. Superior volsella finger-like, 53−63 mm long, 25−27 mm wide, with 9 long setae (Fig. 18). Inferior volsella elongated, 160−170 mm long, with 4 long setae and one well-developed terminal spine. Gonostylus 123−170 mm long, with 9 long setae along inner margin in distal 1/2. HR 0.94−1.47, HV 1.98−2.35.

#### Female, pupa and larva.

Unknown.

#### Type material.

Holotype: Male, CHINA, Zhejiang, Linan City, Qingliangfeng Mountain, 16.v.2012, leg. XL Lin, sweep net. 4 Paratypes: 4 males, same data as holotype.

#### Etymology.

The specific epithet is an adjective referring to the type locality, Linan City.

#### Remarks.

*Stenochironomus linanensis* sp. n. is similar to *Stenochironomus macateei* (Malloch, 1905), *Stenochironomus maculatus* Borkent, 1984 and *Stenochironomus recticaudatus* Borkent, 1984 in the structure of the hypopygium and the inferior volsella with a strong terminal spine, but can be separated from these species by the differences given in Table 4.

**Table 4.** Main differences between *Stenochironomus linanese* sp. n., *Stenochironomus macateei*, *Stenochironomus maculatus* and *Stenochironomus recticaudatus*.

	*Stenochironomus linanese*	*Stenochironomus macateei*	*Stenochironomus maculatus*	*Stenochironomus recticaudatus*
Wing	transparent	transparent	entire wing with pigmentation	transparent
Median setae of tergite IX	10−15	35−37	25−28	35−37
posterior margin of tergite IX	with 12−14 setae and 4 spines	with 8 setae and 6 spines	with 8 setae and 4 spines	with 14 setae and 8 spines
Anal point	apex of anal point slightly swollen and rounded	parallel-sided	apex of anal point slightly swollen and rounded	apex of anal point slightly swollen and rounded
Superior volsella	with 9 setae	with 4−5 setae	with 4−6 setae	with 6 setae
Coloration	whole body yellow, without dark pigmentation	whole body yellow, without dark pigmentation	postnotum, scutum and scutellum with dark pigmentation; other parts of body yellow	dark pigmentation entirely absent except on tarsomeres 3−5 of all legs

#### Distribution.

The species is known from Zhejiang Province of Oriental China.

### Key to males of the genus *Stenochironomus* in China

**Table:** 

1	Inferior volsella with a well-developed terminal spine	**2**
–	Inferior volsella without a well-developed terminal spine	**7**
2	Wing membranes with dark pigmentation	**3**
–	Wing membranes without any pigmentation	**4**
3	Legs almost entire brown, posterior area smoky area between veins C and M pale	***Stenochironomus gibbus* (Fabricius, 1805)**
–	Legs yellow; entire wing smoky gray	***Stenochironomus maculatus* Borkent, 1984**
4	Apex of anal point swollen and rounded	**5**
–	Apex of anal point not swollen and rounded	**6**
5	Superior volsella with 9 setae; posterior margin of tergite IX with 12−14 setae and 4 spines	***Stenochironomus linanensis* sp. n.**
–	Superior volsella with 4 setae; posterior margin of tergite IX with 14−16 setae	***Stenochironomus koreanus* Borkent, 1984**
6	Posterior edge of tergite IX with 8 long setae and 6 spines; anal point parallel-sided	***Stenochironomus macateei* (Malloch, 1915)**
–	Posterior edge of tergite IX with 14 long setae, without any spine; anal point roughly triangular, apically pointed	***Stenochironomus mucronatus* Qi, Shi & Wang, 2008**
7	Wing membranes with dark pigmentation	**8**
–	Wings without any pigmentation or with narrow pigment areas around RM and along veins M_3+4_ and Cu_1_	**10**
8	Abdomen and hypopygium light yellow	***Stenochironomus inalemeus* Sasa, 2001**
–	Abdominal tergites I−IV light yellow, tergites V−VIII light brown, hypopygium dark brown	**9**
9	Preepisternum with brown spots; anal point slender and parallel-sided, apically rounded	***Stenochironomus nubilipennis* Yamamoto, 1981**
–	Preepisternum without any pigmentation; anal point slender and parallel-sided, apically pointed	***Stenochironomus satorui* (Tokunaga & Kuroda, 1936)**
10	Posterior margin of tergite IX with spines	***Stenochironomus brevissimus* sp. n.**
–	Posterior margin of tergite IX without spines	**11**
11	Entire body yellow, without dark pigmentation; wings transparent, without any pigmentation; inferior volsella with 3 long setae	***Stenochironomus hainanus* Qi, Shi & Wang, 2008**
–	Body yellow, with brown spots on thorax, abdomen, hypopygium and legs; wings with narrow pigment areas around RM and along veins M_3+4_ and Cu_1_; inferior volsella with 6 long setae	***Stenochironomus totifuscus* Sublette, 1960**

## Supplementary Material

XML Treatment for Stenochironomus brevissimus

XML Treatment for Stenochironomus linanensis
